# Cardiovascular Health at Age 5 Years: Distribution, Determinants, and Association With Neurodevelopment

**DOI:** 10.3389/fped.2022.827525

**Published:** 2022-04-11

**Authors:** Rachel E. Climie, Muriel Tafflet, Thomas van Sloten, Blandine de Lauzon-Guillain, Jonathan Y. Bernard, Patricia Dargent-Molina, Sabine Plancoulaine, Sandrine Lioret, Xavier Jouven, Marie-Alines Charles, Barbara Heude, Jean-Philippe Empana

**Affiliations:** ^1^Université de Paris, Inserm, U970, Paris Cardiovascular Research Center (PARCC), Integrative Epidemiology of Cardiovascular Disease, Paris, France; ^2^Menzies Institute for Medical Research, University of Tasmanian, Hobart, TAS, Australia; ^3^Sports Cardiology, Baker Heart and Diabetes Institute, Melbourne, VIC, Australia; ^4^Université Paris Cité, Inserm, INRAE, Centre for Research in Epidemiology and StatisticS (CRESS), Paris, France; ^5^Department of Internal Medicine, Cardiovascular Research Institute Maastricht, Maastricht University Medical Centre, Maastricht, Netherlands; ^6^Singapore Institute for Clinical Sciences (SICS), Agency for Science, Technology and Research (A*STAR), Singapore, Singapore

**Keywords:** childhood, determinants, neurodevelopment, primordial prevention, cardiovascular disease

## Abstract

**Background:**

Early childhood may represent an opportune time to commence primordial prevention of cardiovascular disease (CVD, i.e., prevention of risk factors onset), but epidemiological evidence is scarce. We aimed to examine the distribution and parental and early life determinants of ideal cardiovascular health (CVH) in children up to 5 years and to compare the level of cognitive development between children with and without ideal CVH at age 5 years.

**Methods:**

Using data from the Etude sur les déterminants pré et post natals précoces du Développement psychomoteur et de la santé de l'Enfant (EDEN) study, a French population-based mother–child cohort study, CVH was examined in children at 5 years of age based on the American Heart Association CVH metrics (ideal body mass index, physical activity, diet, blood pressure, cholesterol and glucose levels, and passive smoking, considered in sensitivity analysis only). Children were categorized as having ideal (five to six ideal metrics) or non-ideal CVH (<5 ideal metrics). Intelligence quotient (IQ) at age 5 years was assessed using the French version of the Wechsler Preschool and Primary Scale of Intelligence.

**Results:**

Among the 566 children (55% boys), only 34% had ideal CVH. In fully adjusted logistic regression, boys compared to girls (OR = 1.77, 95% CI 1.13–2.78), children with intermediate (1.77, 1.05–2.98) or ideal (2.58, 1.38–4.82) behavioral CVH at age 3 years and children who spent < 30 min/day watching television (1.91, 1.09–3.34) at age 3 years were more likely to have ideal CVH at age 5 years. At age 5 years, there was a significant 2.98-point difference (95% CI 0.64–5.32) in IQ between children with and without ideal biological CVH after adjusting for confounders.

**Conclusion:**

This study highlights that only a third of children aged 5 years had ideal CVH and identified modifiable determinants of ideal CVH and is suggestive of an association between CVH and neurodevelopment at a young age.

## Introduction

Primordial prevention (i.e., the prevention of risk factor onset) is increasingly recognized as a complimentary strategy for the prevention of cardiovascular disease (CVD) but also for an optimal brain health (1, 2). In 2010 the American Heart Association developed a seven-item tool [diet, smoking, physical activity, body mass index (BMI), blood pressure (BP), blood glucose, and cholesterol levels] to monitor and promote ideal cardiovascular health (CVH). Higher CVH in adulthood has been related to lower risk of not only CVD (3–5) but also dementia and cognitive decline (6, 7).

Although overt chronic disease may not appear until the fifth or sixth decade of life, the risk factors begin to develop in childhood or even earlier such as *in utero* (8). Observational data suggest that exposure to poor health-related behaviors in childhood are major contributors to health outcomes later in life (9–15). Therefore, early childhood may represent an opportune time to establish life-long health-promoting habits that can prevent the onset of risk factors and future development of chronic diseases (16–19). In this context, primordial prevention in early life has been recognized to be of primary importance (20, 21). However, the distribution of CVH in early childhood (<6 years of age) is poorly described. One US study examined the distribution of all seven CVH metrics in children aged 4–7 years; another evaluated the distribution of four specific metrics in children aged 2–11 years (22, 23). The determinants of ideal CVH at a young age have been investigated in one US study which focused on pre- and peri-natal characteristics (22). Furthermore, the early years of life are a period of rapid neurological development (24), but the potential association between CVH and neurodevelopment in early childhood has not been yet investigated.

We examined the distribution and parental and early life determinants of ideal CVH in children up to 5 years of age. We also explored the potential association between CVH and cognitive development in children at this age.

## Methods

### Study Participants and Overview

The EDEN (Etude sur les déterminants pré et post natals précoces du Développement psychomoteur et de la santé de l'Enfant) study is an ongoing French mother–child cohort study involving 2,002 pregnant women recruited before 24 weeks of gestation from two maternity wards (Nancy vs. Poitiers) in France between September 2003 and January 2006 (25). The study design involved repeated in-person clinical examinations of the mother and child, along with questionnaires relating to parental and child health and lifestyle. Written informed consent was obtained from all mothers at inclusion and for the child after delivery. The EDEN study was approved by the Ethics Committee of the Kremlin Bicêtre Hospital and by the Commission Nationale de l'Informatique et des Libertés [National Committee for Processed Data and Freedom (CNIL)].

### Childhood Data

#### CVH Metrics

The definition of the seven CVH metrics is detailed in the Supplementary Material. The cutoff value of ≥180 min/day (average daily time of outdoor games and walking) was used to define ideal physical activity, and seasonal variability in physical activity was accounted for (26). Ideal diet was defined as meeting two of the three following conditions: >4.5 fruits and vegetables/day, more than twice per week of fish, sugar-sweetened beverages <4 times/week, as data on fiber-rich aliments or sodium intake were not available. Ideal BMI was defined as a BMI lower than age- and sex-specific threshold for overweight as defined by the International Obesity Task Force (27). Given the age of the population, ideal smoking was defined as not exposed to parental smoking (i.e., passive smoking) and was therefore considered in a sensitivity analysis. Ideal BP was categorized as an age-, height-, and sex-specific systolic BP <90th percentile and diastolic BP <90th percentile within each of the two EDEN Study centers. Ideal fasting total cholesterol and glucose levels were defined as <5.55 and <4.40 mmol/L, respectively (28).

Ideal CVH status was defined as the presence of five to six metrics at the ideal level in main analysis (1). The number of metrics at the ideal level (range 0–6 in main analysis) was also considered. Poor, intermediate, and ideal behavioral or biological CVH was defined by the presence of zero to one, two, and three corresponding metrics at the ideal level in main analysis (Supplementary Table S1).

#### Other Childhood Data

Information on birth weight, length, gestational age, breastfeeding initiation, and parity was obtained at delivery, and birth weight z-score of the child was defined based on gestation age, birth length, pre-pregnancy maternal weight, and parity. The number of hours slept overnight and number of hours spent watching television daily were also obtained via parental questionnaires at 3 and 5 years of age.

#### Cognitive Development

The French version of the Wechsler Preschool and Primary Scale of Intelligence (29) was administered to children at 5 years of age by a trained psychologist at each study center. Age-adjusted verbal, performance, and total intelligence quotient (IQ) were calculated based on information, vocabulary, word reasoning, block design, matrix reasoning, picture concepts, and coding subtests.

### Parental Data

The CVH metrics of the mother prior to pregnancy are defined in Supplementary Table S2, and other parental data collected during and after pregnancy are detailed in the Supplementary Material.

### Statistical Analysis

Comparisons between groups were performed using t-tests and chi-square tests where appropriate. The determinants of ideal CVH status at 5 years of age were investigated using a multivariable binary logistic regression analysis with sequential adjustment accounting for (1) parental and socio-demographic characteristics (i.e., study centers, age of the parents, family education level, mother living alone status, and mother country of birth); (2) maternal characteristics and behavior prior to and during pregnancy (i.e., hypertension status prior to pregnancy, behavioral CVH prior to pregnancy, comorbidities during pregnancy, depressive symptoms during pregnancy, smoking status during pregnancy, alcohol consumption during pregnancy, parity, and father BMI); (3) child birth data (i.e., sex of the baby, gestational age, birth weight, and breastfeeding status) and; (4) behavioral data of children at 3 years of age (behavioral CVH status, overnight sleep duration, and daily time spent watching TV). With this approach, the relevance of a variable is determined in the model in which the variable was first entered, without considering its performance in subsequent models. Multivariable ordinal logistic regression analysis was performed to identify factors associated with the number of metrics at ideal level (range 0–6 in main analysis). The association of ideal CVH status and number of ideal CVH metrics with IQ were analyzed using multivariable linear regression analysis. This analysis was adjusted for *a priori* defined confounders from the literature that include study center, maternal age, maternal pre-pregnancy BMI, depression during pregnancy, smoking and alcohol consumption during pregnancy, parity, child's sex, birth weight z-score, gestational age, breastfeeding, parental education, household income, family stimulation score, and time spent watching television at age 5 years.

In sensitivity analysis, analyses were repeated after including the smoking metric in the definition of CVH at 5 years of age while keeping the same definition of ideal CVH status (at least five ideal metrics). Analyses were performed with SAS 9.4 (SAS Institute Inc., Cary, North Carolina, USA NC); all *p*-values were two sided, and *p* < 0.05 was considered statistically significant.

## Results

### Analytical Sample

**Figure 1:**
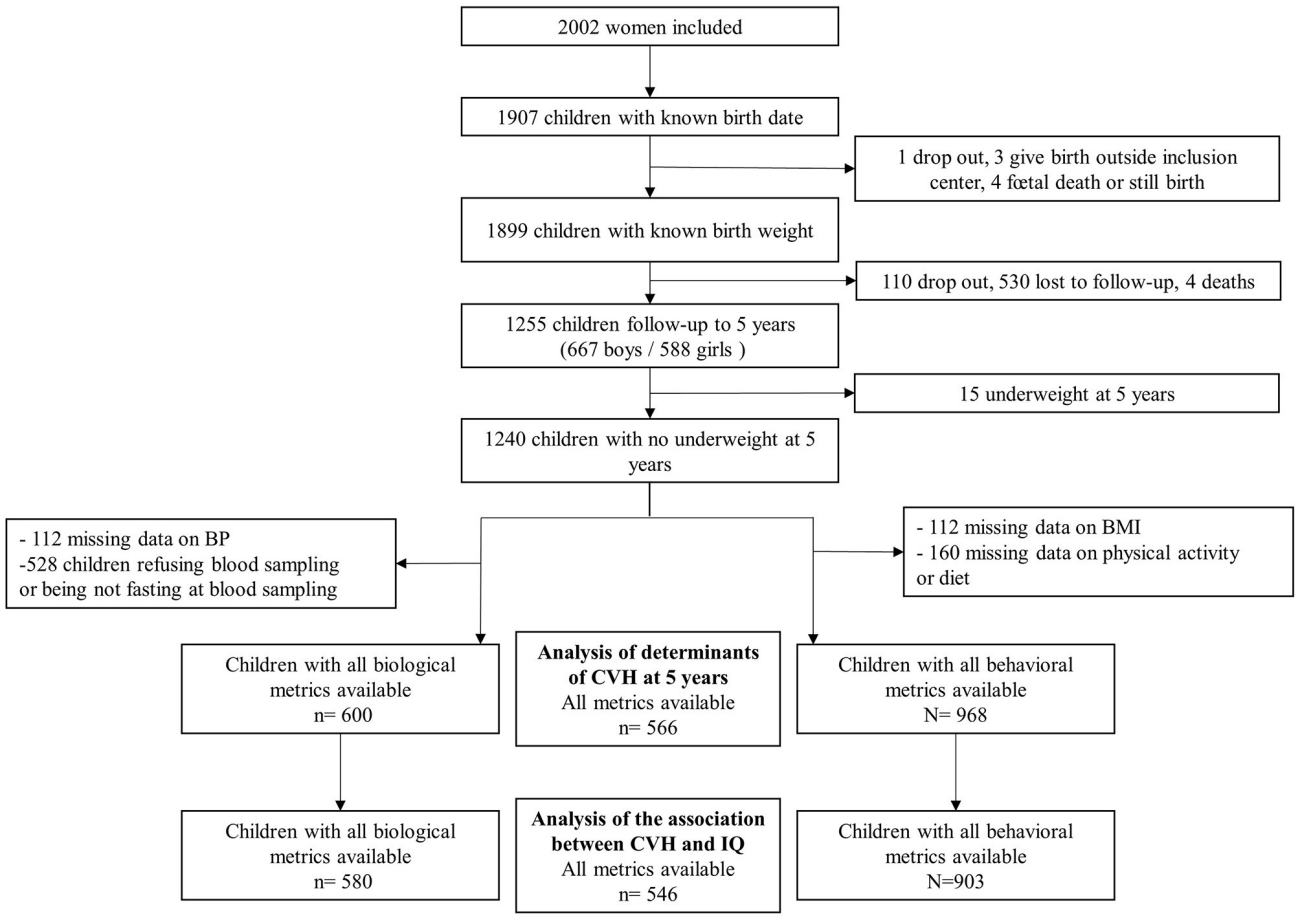
Study flowchart. CVH, cardiovascular health; IQ, intelligence quotient.

The study flow chart of the population is described in Figure 1. Of the 1,899 children who were born alive and had a known birth weight, 1,255 attended the 5-year follow up assessment. Among them, 968 had complete data for behavioral CVH metrics, and 600 had complete data for biological CVH metrics (528 children refused blood samples or were not in fasting state), resulting in 566 with complete data for all CVH metrics. The early life characteristics (Supplementary Table S3) together with the parental characteristics (Supplementary Table S4) of those included compared to those excluded did not differ, except that ideal diet was less often observed in the excluded children.

### Distribution of Ideal CVH in Children at 5 Years of Age

**Figure 2:**
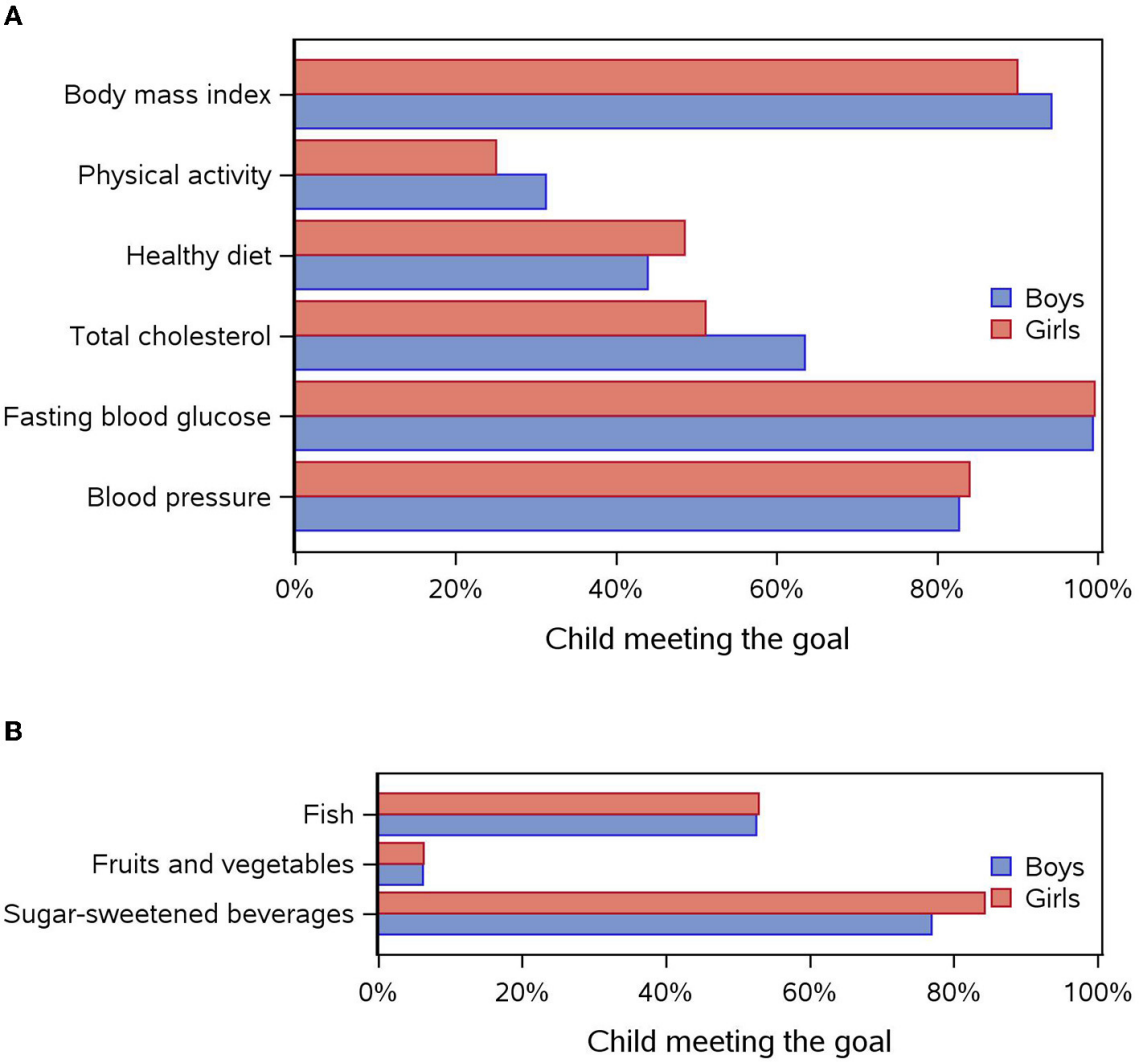
**(A)** Distribution of each cardiovascular health metric (meeting the goal) according to sex. **(B)** Distribution of each component of the diet metric (meeting the goal) according to sex.

Overall, 34% of children (*n* = 195) had at least five metrics at the ideal level, of whom 39% were boys and 29% girls (*p* = 0.02). In addition, 6% had six metrics at the ideal level (7% of boys and 5% of girls). Boys were more likely than girls to have ideal levels of BMI (*p* = 0.008), physical activity (*p* = 0.03), and total cholesterol levels (*p* = 0.001) (Figure 2A), whereas girls were more likely to have ideal sugar-sweetened beverage consumption (*p* = 0.001, Figure 2B).

### Early Life and Parental Characteristics in Children With and Without Ideal CVH at 5 Years Age

Apart from sex (*p* = 0.023), other birth and neonatal characteristics did not differ between children with and without ideal CVH at 5 years of age (Table 1). Ideal levels of BMI, diet, fish, and sugar-sweetened beverage consumption at age 3 years were more likely observed in children with ideal compared to non-ideal CVH, at 5 years of age (*p* < 0.001).

**Table 1:** Early life characteristics in children with and without ideal cardiovascular health (CVH) at 5 years of age.

	**Ideal CVH^a^ at 5 years of age** ***n*** **= 195**	**Non-ideal CVH at 5 years of age** ***n*** **= 371**	***p*** **-value**
**Characteristics at birth**			
Study center (Nancy vs. Poitiers)	113 (58)	213 (57)	0.90
Male sex	121 (62)	193 (52)	0.02
Preterm	8 (4)	21 (6)	0.42
Gestational age, weeks (mean ± SD)	39 ± 2	39 ± 2	0.37
Birth weight, g (mean ± SD)	3,334 ± 447	3,264 ± 550	0.27
**Characteristics after birth**			
Breastfed	148 (76)	272 (74)	0.54
Time spent breastfeeding, months (mean ± SD)	3 ± 4	3 ± 4	0.86
**Characteristics at 3 years age**			
Ideal level of blood pressure	162 (86)	283 (84)	0.50
Ideal level of body mass index	182 (100)	305 (92)	<0.001
Ideal diet	109 (59)	138 (41)	<0.0001
Ideal consumption of fish	112 (61)	163 (49)	0.008
Ideal consumption of fruit and vegetables	12 (7)	15 (5)	0.32
Ideal consumption of sugar-sweetened beverage	168 (91)	273 (82)	0.003
Ideal level of physical activity	75 (42)	120 (37)	0.23
Behavioral CVH^b^			
*Poor*	43 (25)	142 (45)	<0.0001
*Intermediate*	86 (50)	126 (40)	
*Ideal*	42 (25)	48 (15)	
Time spent watching television daily			
* ≤ 30 min*	54 (29)	84 (25)	0.26
*>30 min to 1 h*	58 (31)	128 (39)	
*>1 h*	70 (38)	116 (35)	
Overnight sleep duration, h (mean ± SD)	11 ± 0.6	11 ± 0.6	0.57
**Characteristics at 5 years of age**			
Age, years (mean ± SD)	5.6 ± 0.2	5.6 ± 0.1	0.09
Weight, kg (mean ± SD)	20 ± 2	20 ± 3	0.73
Height, cm (mean ± SD)	114 ± 40	114 ± 5	0.44
Time spent watching television daily			
≤ *30 min*	24 (13)	40 (11)	0.67
*30 min to 1 h*	61 (32)	108 (3)	
*>1 h*	105 (55)	214 (59)	
Overnight sleep duration, h (mean ± SD)	11 ± 0.5	11 ± 0.5	0.30

*Data are n (%) unless otherwise stated. Biological CVH (blood pressure, total cholesterol, and fasting glycemia) at age 3 years could not be computed as blood was only taken at age 5 years*.

*Some data on early life characteristics were missing in children, ranging from n = 79 for behavioral CVH in children at age 3 years to n = 1 for breastfeeding*.

a *Overall cardiovascular health (CVH) at age 5 years was computed according to the level of six metrics including body mass index, physical activity, diet, blood pressure, total cholesterol, and fasting glycemia (passive smoking was considered in sensitivity analysis only); those children with at least five out of the six metrics at the ideal level were considered to have an ideal overall CVH*.

b *Behavioral CVH at age 3 years was computed according to the level of body mass index, diet, and physical activity. Those children with zero to one, two, and three behavioral metrics at the ideal level were considered to have a poor, intermediate, and ideal behavioral CVH status, respectively*.

Children who had ideal CVH at age 5 years were more likely to have a mother who had consumed the ideal level of fish and fruit and vegetables prior to pregnancy, compared to mothers who did not (*p* = 0.014 and 0.015, respectively, Supplementary Table S5).

### Determinants of Ideal CVH in Children at 5 Years of Age

After adjusting for socio-economic factors (level 1), comorbidities and behavior of the mother prior to birth and BMI of the father (level 2), birth data (level 3), and behavioral data of children at age 3 years (level 4), boys compared to girls (OR = 1.77; 95% CI: 1.13; 2.78), children with intermediate (1.77; 1.05; 2.98) or ideal (2.58; 1.38; 4.82) behavioral CVH at age 3 years, and children who spent <30 min/day watching television at age 3 years (1.91;1.09; 3.34) were more likely to have ideal CVH at age 5 years (Table 2). When considering the number of metrics at the ideal level as the outcome, sleep duration at age 3 years was an additional determinant of ideal CVH at 5 years of age (Supplementary Table S6).

**Table 2:** Determinants of ideal cardiovascular health in children at 5 years of age.

	**Determinants**	**Model 1**		**Model 2**		**Model 3**		**Model 4**	
		**OR (95%CI)**	***p*** **-value**	**OR (95%CI)**	***p*** **-value**	**OR (95%CI)**	***p*** **-value**	**OR (95%CI)**	***p*** **-value**
Level 1	Study center (Nancy *vs*. Poitiers)	1.01 (0.70; 1.44)	0.97	0.89 (0.59; 1.34)	0.56	0.88 (0.57; 1.35)	0.57	0.89 (0.54; 1.48)	0.65
	Age of mother (years)	0.98 (0.93; 1.03)	0.45	0.95 (0.90; 1.01)	0.08	0.95 (0.89; 1.00)	0.07	0.94 (0.88; 1.01)	0.10
	Age of father (years)	1.00 (0.96; 1.05)	0.92	1.03 (0.98; 1.08)	0.25	1.03 (0.98; 1.08)	0.24	1.03 (0.98; 1.09)	0.29
	Family educational level								
	*Secondary vs. high school*	0.98 (0.52; 1.82)	0.94	1.06 (0.54; 2.07)	0.86	1.03 (0.52; 2.03)	0.93	0.92 (0.39; 2.19)	0.85
	*Tertiary vs. high school*	0.87 (0.52; 1.45)	0.60	0.81 (0.46; 1.43)	0.47	0.75 (0.42; 1.35)	0.34	0.69 (0.33; 1.46)	0.33
	Mother living alone when child 5 years of age	0.85 (0.40; 1.80)	0.67	0.72 (0.32; 1.63)	0.42	0.67 (0.29; 1.55)	0.35	0.45 (0.15; 1.38)	0.16
	Mother born in France	1.43 (0.76; 2.70)	0.27	0.94 (0.39; 2.27)	0.89	1.19 (0.47; 3.04)	0.72	1.38 (0.38; 5.03)	0.62
Level 2	Hypertension prior to pregnancy			1.68 (0.49; 5.72)	0.41	1.86 (0.54; 6.40)	0.33	2.78 (0.67; 11.48)	0.16
	Behavioral cardiovascular health prior to pregnancy^a^								
	*Intermediate vs. poor*			1.26 (0.75; 2.14)	0.38	1.34 (0.78; 2.30)	0.29	1.57 (0.82; 3.00)	0.17
	*Ideal vs. poor*			1.22 (0.69; 2.15)	0.49	1.27 (0.71; 2.28)	0.41	1.53 (0.77; 3.03)	0.22
	Comorbidities that developed during pregnancy^b^			1.20 (0.65; 2.20)	0.56	1.25 (0.67; 2.32)	0.48	1.13 (0.54; 2.40)	0.74
	Depressive symptoms during pregnancy, CESD ≥ 23			0.88 (0.40; 1.90)	0.73	0.90 (0.41; 1.98)	0.80	1.19 (0.46; 3.07)	0.72
	Smoking during pregnancy			0.96 (0.57; 1.61)	0.89	0.96 (0.57; 1.62)	0.87	0.85 (0.45; 1.62)	0.63
	Alcohol consumption during pregnancy								
	*0–2 vs. no glasses/week*			0.79 (0.52; 1.20)	0.27	0.76 (0.49; 1.16)	0.20	0.66 (0.40; 1.10)	0.11
	*≥2 vs. no glasses/week*			1.39 (0.70; 2.76)	0.34	1.33 (0.66; 2.71)	0.43	1.35 (0.61; 3.01)	0.46
	Primiparous			0.91 (0.60; 1.38)	0.66	0.91 (0.59; 1.38)	0.65	0.79 (0.48; 1.28)	0.34
	Father ideal body mass index			1.19 (0.81; 1.74)	0.38	1.21 (0.82; 1.78)	0.34	1.20 (0.76; 1.90)	0.44
Level 3	Sex (boy vs. girl)					1.60 (1.09; 2.34)	0.02	1.77 (1.13; 2.78)	0.01
	Gestational age					1.10 (0.97; 1.25)	0.14	1.04 (0.90; 1.21)	0.58
	Birth weight z-score					1.08 (0.90; 1.29)	0.43	0.99 (0.80; 1.22)	0.92
	Ever breastfed vs. never breastfed					1.01 (0.64; 1.59)	0.98	1.32 (0.76; 2.27)	0.33
Level 4	Behavioral cardiovascular health at 3 years of age^c^								
	*Intermediate vs. poor*							1.77 (1.05; 2.98)	0.03
	*Ideal vs. poor*							2.58 (1.38; 4.82)	0.003
	Overnight sleep duration (h)							0.80 (0.55; 1.15)	0.22
	Watching television								
	* ≤ 30 min vs. 30 min to 1 h/day*							1.91 (1.09; 3.34)	0.02
	*>1 h vs. 30 min to 1 h/day*							1.18 (0.68; 2.05)	0.55

*Multivariate logistic regression of ideal cardiovascular health status compared to non-ideal cardiovascular health status was performed by adding several variables in blocks. Model 1 is adjusted for socio-demographic factors, model 2 is adjusted for model 1 + the mothers pregnancy data, model 3 is adjusted for model 2 + birth data, and model 4 is adjusted for model 3 + relevant cardiovascular health data of the children at 3 years of age*.

*Overall cardiovascular health (CVH) at age 5 years was computed according to the level of six metrics including body mass index, physical activity, diet, blood pressure, total cholesterol, and fasting glycemia (passive smoking was considered in sensitivity analysis only; see Supplementary Table S7); those children with at least five out of the six metrics at the ideal level were considered to have an ideal overall CVH. Biological CVH (blood pressure, total cholesterol, and fasting glycemia) at age 3 years could not be computed as blood was only taken at age 5 years*.

*CESD, French version of the Center for Epidemiologic Studies–Depression (see ref #8, 9 in the Supplementary Material). A score ≥ 23 is indicative of a high likelihood of clinical depression. Birth weight z-score was defined based on gestation age, height, maternal weight, and parity*.

a *Behavioral CVH status in the mother prior to pregnancy was computed according to the smoking status, the level of body mass index, diet, and physical activity. Mothers with zero to one, two, and three to four behavioral metrics at the ideal level were considered to have a poor, intermediate, and ideal behavioral CVH status, respectively*.

b *Hypertension or gestational diabetes*.

c *Behavioral CVH status at age 3 years was computed according to the level of body mass index, diet, and physical activity. Those children with zero to one, two, and three behavioral metrics at the ideal level were considered to have a poor, intermediate, and ideal behavioral CVH status, respectively*.

### Cross-Sectional Association of CVH and Neurodevelopment at 5 Years of Age

In multivariable linear regression analysis, there was a significant difference (β = 2.98, 95% CI: 0.64; 5.32) in IQ performance between children with ideal biological CVH compared to those without ideal biological CVH. There was also a significant difference (β = 2.48, 95% CI: 0.59; 4.36) in IQ performance per increase in the number of ideal biological metrics (Table 3).

**Table 3:** Cross-sectional association between cardiovascular health and intelligence quotient (IQ) in children at 5 years of age.

	**Total IQ**		**Verbal IQ**		**Performance IQ**	
	**β (95%CI)**	***p*** **-value**	**β (95%CI)**	***p*** **-value**	**β (95%CI)**	***p*** **-value**
**Overall cardiovascular health**						
Ideal vs. non-ideal status^a^	0.01 (−2.37; 2.39)	0.99	0.01 (−2.51; 2.52)	0.99	−0.05 (−2.57; 2.47)	0.97
Number of ideal metrics (range 0–6): per 1 unit increase	1.16 (−0.11; 2.43)	0.07	0.94 (−0.41; 2.29)	0.17	1.01 (−0.35; 2.38)	0.14
**Behavioral cardiovascular health**						
Ideal *vs*. non-ideal status^b^	−0.80 (−3.54; 1.94)	0.56	0.77 (−2.12; 3.66)	0.59	−2.12 (−5.05; 0.81)	0.16
Number of ideal metrics (range 0–3): per 1 unit increase	0.49 (−0.75; 1.73)	0.43	1.32 (0.01; 2.62)	0.050	−0.60 (−1.90; 0.73)	0.38
**Biological cardiovascular health**						
Ideal *vs*. non-ideal status^c^	1.62 (−0.62; 3.86)	0.16	−0.18 (−2.54; 2.18)	0.88	2.98 (0.64; 5.32)	0.013
Number of ideal metrics (range 0–3): per 1 unit increase	1.62 (−0.19; 3.42)	0.080	0.23 (−1.67; 2.13)	0.81	2.48 (0.59; 4.36)	0.010

*Multivariate linear regression analysis adjusted for study center, maternal age, maternal pre-pregnancy body mass index, depression during pregnancy, smoking and alcohol consumption during pregnancy, parity, child's sex, birth weight z-score, gestational age, breastfeeding, parental education, household income, family stimulation score, and time spent watching television at age 5 years*.

*Age-adjusted composite scores for verbal, performance, and total intelligence quotient (IQ) were calculated based on information obtained from the French version of the Wechsler Preschool and Primary Scale of Intelligence (27)*.

a *Children with five or six metrics (body mass index, physical activity, diet, blood pressure, total cholesterol, and fasting glycemia; passive smoking was considered in sensitivity analysis only; see Supplementary Table S8) at the ideal level at age 5 years*.

b *Children with three behavioral metrics (body mass index, physical activity, and diet; passive smoking was considered in sensitivity analysis only; see Supplementary Table S8) at the ideal level at age 5 years*.

c *Children with three biological metrics (blood pressure, total cholesterol, and fasting glycemia) at the ideal level at age 5 years*.

### Sensitivity Analysis

When adding the smoking metric to the definition of CVH at age 5 years, 56% of children were not exposed to parental smoking; 61% had at least five metrics at the ideal level, and 3% had seven metrics at the ideal level. In addition to the determinants identified in the main analysis, mothers living alone (inverse association) and absence of parental smoking (positive association) were also associated with ideal CVH at age 5 years (Supplementary Table S7). As in the main analysis, biological CVH was related to performance IQ, and behavioral CVH was additionally associated with verbal IQ (Supplementary Table S8).

## Discussion

Only 34% of the children at 5 years of age had at least five CVH metrics at the ideal level. After accounting for parental and early life characteristics, being a boy, having intermediate or ideal behavioral CVH, and watching < 30 min daily of television at age 3 years were associated with ideal CVH at age 5 years. Higher biological CVH was related to higher performance IQ at 5 years of age.

To our best knowledge, only one other study, the Healthy Start Study in the US (22), has explored the distribution and determinants of all seven CVH metrics in early childhood. The National Health and Nutrition Examination Study (NHANES) reported the prevalence of four CVH metrics (BMI, diet, cholesterol, and BP levels) in US children aged 2–11 years, although BMI was the only metric examined in children <5 years (23). While the Healthy Start Study focused on pre- and peri-natal determinants of CVH, our study additionally examined exposures at 3 years of age. Further, this is the first study to evaluate the association between CVH and brain health in early childhood. Interestingly, the distribution of the individual metrics in the Healthy Start Study and the EDEN study were very similar, even if the prevalence of ideal physical activity and ideal diet was slightly higher in the EDEN study. The distribution of ideal cholesterol and ideal BP was comparable between NHANES and EDEN, but large differences existed regarding the prevalence of ideal BMI and ideal diet; in particular, a mere 0–0.1% had ideal diet in NHANES compared to 40–42% in the current study, and no child had ideal levels of all four metrics in NHANES compared to 24% in our study.

The analysis of the determinants of ideal CVH indicates that parental health and characteristics were not related to CVH of the children at 5 years of age, likely due to the homogenous sample. In the Healthy Start Study, maternal overweight was inversely related to ideal CVH in the children, potentially reflecting the multi-ethnic nature of the cohort, and the mother–child pairs included had lower household income compared to those excluded (22). In the present study, when including passive smoking in the definition of CVH, mothers not living alone and absence of smoking during pregnancy were related to higher ideal CVH in children at age 5 years. This suggests that a favorable family environment is related to ideal CVH in early childhood, in agreement with results of the Healthy Lifestyle in Europe by Nutrition in Adolescence (HELENA) study which showed that higher maternal education was related to higher CVH in adolescents (30). Furthermore, childbirth data were not related to CVH at age 5 years, possibly due to the inclusion of relatively healthy babies. Contrary to what is observed in adulthood (31), boys were almost twice as likely to have ideal CVH compared to girls. This may be explained in part by a persistently observed gender-based disparity in physical activity levels among children, whereby girls are typically less physically active than boys (32, 33). The higher level of physical activity observed in boys may also explain why boys also had lower levels of cholesterol levels compared to girls (34). This is consistent with the results of the Healthy Start Study, in which boys were twice as likely (OR = 2.14; 95% CI: 1.22–3.65) to have at least six ideal metrics (22). We also observed that children with intermediate or ideal behavioral CVH at age 3 years were significantly more likely to have ideal CVH at age 5 years, confirming that healthy behaviors that develop early on in life track into older childhood. Interestingly, watching < 30 min/day of television at age 3 years was positively related to higher CVH at 5 years, similarly to the results from the HELENA study (30). Together with the evidence that only 15% of CVH is inherited (35), the present findings emphasize that ideal CVH in early childhood is mostly determined by behavioral (i.e., modifiable) factors.

Higher biological CVH at 5 years of age (ideal level of BP, total cholesterol, and fasting glycemia) was associated with higher performance IQ. This is in line with results from the Young Finns study showing that cumulative exposure to BP and total cholesterol from 3 to 18 years of age is related to cognition in middle age (36). From a biological perspective, high BP in adults may lead to cerebral microvascular dysfunction and contribute to blood–brain barrier leakage, impaired neurovascular coupling, and disturbed capillary flow patterns leakage (37). Furthermore, animal data suggest that higher cholesterol is related to neuro-inflammatory changes, enhanced cortical beta-amyloid and Tau (38), and cerebral blow flow impairment (39). Whether these mechanisms are at play in early childhood remains to be determined. This finding is also consistent with studies in adults indicating that higher CVH is associated with lower cognitive decline and dementia risk later in life (6, 7). On the other hand, the association between behavioral CVH and verbal IQ was only borderline significant. BMI (40), diet (41), and physical activity levels (42) in early childhood have been related to cognition in childhood; however, measurement errors by the parents reporting the children's diet and physical activity, inclusion of homogeneous healthy children, and the relatively small sample size of the study may explain the insignificant result.

The current findings have important public health implications. Firstly, only 34% of the children at age 5 years had at least five metrics at the ideal level, indicating that promoting primordial prevention in early childhood should be a priority. This is a multilevel process involving the children themselves and also their families and schools, together with public health policies on several aspects including the living environment, access to health care, or taxations (18). Secondly, apart from sex, early life (i.e., at 3 years of age) determinants of ideal CVH at age 5 years were modifiable, highlighting the need to establish healthy behaviors from a young age. Indeed, when healthy behaviors are established early on in life, they are more likely to be adopted in adulthood (43). In relation to physical activity, the World Health Organization (44) recommends that children < 5 years of age should engage in a variety of activities and include moderate to vigorous intensity. Thirdly, the current findings support the clinical relevance of promoting higher CVH in early childhood by suggesting an association between CVH and optimal brain health.

### Limitations

Biological CVH metrics were missing in nearly half of the study population, mainly due to not being in a fasted state or refusing to have blood taken, which may have affected the statistical power of some analyses and the precision in the estimates. Additionally, there may have been other determinants of CVH that were not included such as adverse childhood experiences (i.e., exposure to violence, stress, or crime) or environment factors (pollution, built environment, or exposure to noise). Furthermore, the reported cross-sectional association between CVH and IQ should not be interpreted as causal and needs to be confirmed in future prospective analysis. The use of complimentary methods to assess neurodevelopment should also be confirmed in future studies, as IQ only measures certain dimensions of cognition. Finally, the EDEN population is mostly composed of urban, well-educated, and high-income households and Caucasian participants, and therefore, the current results may not apply to other populations.

## Conclusion

This study highlighted that only a third of children aged 5 years had ideal CVH, identified early life and modifiable determinants of ideal CVH at 5 years of age, and is suggestive of an association between higher CVH and neurodevelopment in early childhood.

## Data Availability Statement

The raw data supporting the conclusions of this article will be made available by the authors, without undue reservation.

## Ethics Statement

Written informed consent was obtained twice from parents, once at enrolment and once after the child's birth. The study was approved by the Ethics Research Committee (Comité Consultatif de Protection des Personnes dans la Recherche Biomédicale) of the Bicêtre Hospital and by the Data Protection Authority (Commission Nationale de l'Informatique et des Libertés). All research was performed in accordance with relevant guidelines and regulations.

## Author Contributions

RC: methodology, writing—original draft, and funding acquisition. TS and XJ: writing—review and editing. BL-G, JB, PD-M, SP, MT, and SL: formal analysis and writing—review and editing. M-AC: methodology, formal analysis, conceptualization, and design of the EDEN study. BH and J-PE: conceptualization, methodology, resources, supervision, and project administration. All authors contributed to the article and approved the submitted version.

## Funding

The EDEN study was supported by the Foundation for Medical Research (FRM), National Agency for Research (ANR), National Institute for Research in Public Health (IRESP: TGIR cohorte santé 2008 program), French Ministry of Health (DGS), French Ministry of Research, INSERM Bone and Joint Diseases National Research (PRO-A), Human Nutrition National Research Programs, Paris-Sud University, Nestlé, French National Institute for Population Health Surveillance (InVS), French National Institute for Health Education (INPES), the European Union FP7 programs (FP7/2007–2013, HELIX, ESCAPE, ENRIECO, Medall projects), Diabetes National Research Program [through a collaboration with the French Association of Diabetic Patients (AFD)], French Agency for Environmental Health Safety (now ANSES), Mutuelle Générale de l'Education Nationale a Complementary Health Insurance (MGEN), French National Agency for Food Security, and the French-Speaking Association for the Study of Diabetes and Metabolism (ALFEDIAM). This project was supported by funding from the European Union's Horizon 2020 research and innovation program under grant agreement 874739 (LongITools). RC was supported by a Postdoctoral Fellowship from the National Heart Foundation of Australia (Award ID: 102484). J-PE received support from the non-profit Fondation Française de Cardiologie to run this project.

## Conflict of Interest

The authors declare that the research was conducted in the absence of any commercial or financial relationships that could be construed as a potential conflict of interest.

## Publisher's Note

All claims expressed in this article are solely those of the authors and do not necessarily represent those of their affiliated organizations, or those of the publisher, the editors and the reviewers. Any product that may be evaluated in this article, or claim that may be made by its manufacturer, is not guaranteed or endorsed by the publisher.
