# Supplementary material for: Cardiovascular Health at Age 5 Years: Distribution, Determinants, and Association With Neurodevelopment
Source: Front Pediatr. 2022 Apr 11;10:827525. doi: 10.3389/fped.2022.827525 (PMC9035843; doi:10.3389/fped.2022.827525)
Supplement: Supplementary file 1 [file Data_Sheet_1.docx]

Supplementary Material

# Table of Contents

# Supplementary Methods

# STable1. Definition of ideal cardiovascular health metrics at three and five years of age.

# STable2. Definition of ideal behavioral cardiovascular health metrics in the mother prior to pregnancy.

# STable3. Early life characteristics of included and excluded children among those followed up until five years of age.

STable4. Parental characteristics of included and excluded study participants among those followed up until five years of age.

# STable5. Parental characteristics in children with and without ideal cardiovascular health at five years age.

# STable6. Determinants of number of cardiovascular health metrics at ideal level in children at five years of age.

STable7. Determinants of ideal cardiovascular health status in children at five years of age after including passive smoking in the definition of cardiovascular health.

# STable8. Cross-sectional association between cardiovascular health and intelligence quotient in children at five years of age after including passive smoking in the definition of cardiovascular health.

Supplementary references.

# Supplementary Methods

**EDEN study design**

The EDEN (Etude sur les déterminants pré et post natals précoces du Développement psychomoteur et de la santé de l’Enfant) mother-child cohort is an ongoing French observational cohort study involving 2002 pregnant women recruited before 24 weeks of gestation from two maternity wards (Poitiers and Nancy University hospitals, France) between September 2003 and January 2006 (1). Birth data were obtained from 1899 mother-infant pairs. At the five-year follow-up, data were available for 1255 (66%) mothers and children. The exclusion criteria included twin pregnancies, known diabetes before pregnancy, illiteracy and intention to move outside the region within the next three years.

**Definition of the CVH metrics in the children**

The definition of each metric is summarized in eTable 1. Physical activity at three and five years of age was defined using a questionnaire completed by a parent. Physical activity was assessed from three questions relating to the time (minutes per day) the child usually spent playing outdoors (e.g., in a backyard, a park, a playground) on a typical weekday (excluding Wednesday), on Wednesday (which was a day off at the time of the survey) and on weekend days. The average daily time was calculated as the sum of typical weekday (multiplied by four), typical Wednesday and typical weekend television time (multiplied by two), with the total divided by seven (2). The cut off value of ≥180 min/day was used to define ideal physical activity at age three and five (2). As the questionnaire was completed at various times throughout the year and physical activity varies according to season, we selected the percentile of the distribution of physical activity in spring corresponding to 180 minutes/day, and applied this percentile to other seasons (2). The 71^th^ percentile of the distribution of physical activity in spring corresponded to 180min/day (all children below this were considered non-ideal), which corresponded to 162 minutes/day in other seasons. Diet was assessed using a Food Frequency Questionnaire (FFQ) completed by a parent when the children were three and five years of age (3). The FFQ included 26 common food groups along with seven possible responses ranging from “never” to “several times per day”, that were converted into weekly frequencies. Frequency of fruit, vegetable, sugar sweetened beverage and fish consumption were available in the EDEN study but data on fiber rich aliments or sodium intake were not. Children were considered to have ideal diet if they met two of the three following conditions: >4.5 fruits and vegetables/day, more than twice per week of fish, sugar sweetened beverages <4/week. Height and weight were obtained via clinical examination and body mass index (BMI) was determined. BMI was considered as ideal, when below the age- and sex-specific threshold for overweight as defined by the International Obesity Task Force (4). Ideal smoking was defined as unexposed to parental smoking (i.e. passive smoking) at age five and was considered in a sensitivity analysis only. Blood pressure (BP) was measured in triplicate using an appropriately sized cuff and an oscillometric COLIN 8800 C device, after the child had been resting supine, silently for five minutes, and following a standardized protocol in both centers. BP was categorized as ideal systolic BP <90th percentile and diastolic BP <90th percentile height, age, sex and center specific within the EDEN population (5). Ideal fasting total cholesterol and glucose levels were defined as <5.55mmol/L and <4.40mmol/L, respectively (6).

**Parental data**

*Pre-pregnancy data*

Weight was self-reported prior to pregnancy. Smoking was self-reported. Physical activity was assessed using the validated Baecke questionnaire (7) during the first trimester and was used as a proxy for pre-pregnancy physical activity level. Diet was assessed via a food frequency questionnaire.

*During pregnancy data*

Maternal height was measured with a wall stadiometer (Hamburg, Germany) to the nearest 0.2 cm. BMI was calculated based on pre-pregnancy weight and height measured during pregnancy. Hypertension and gestational diabetes were collected during a clinical examination. Mothers attended the examination in a fasted state and received a 50-g glucose load, and glucose concentrations were measured 1 h after the glucose challenge. Women with plasma glucose >130mg/dl in Nancy and 140mg/dl in Poitiers were scheduled for a 100-g three hours oral glucose tolerance test. Finally, clinical diagnoses of gestational diabetes were extracted from clinical records, and the difference in thresholds between the two centers did not result in different gestational diabetes prevalence. Alcohol consumption was defined as no alcohol, less than 2 glasses per week, 2 glasses or more. Depressive symptoms were assessed using the French version of the Center for Epidemiologic Studies–Depression (CESD) (8). A score equal or greater than 23 has been validated to indicate a high likelihood for depression (8, 9).

*Paternal data*

Paternal height and weight were measured in the father at inclusion into the study.

*Sociodemographic data*

Sociodemographic data information was collected via self-report questionnaire. Education level of the mother and father were collected at inclusion and classified as high school (year 10 completion), secondary (year 12 completion) and tertiary (bachelor’s degree), with the highest level obtained between the couple representing the family education level. Household income was determined during the pregnancy and every year thereafter. During the clinical visit at 5-6 years of age, the French Home Observation for the Measurement of the Environment Inventory-Short Form (10) was administered providing information pertaining to the home environment of the child (language stimulation, learning stimulation and variety in experience, family stimulation score).

# STable1. Definition of ideal cardiovascular health metrics at three and five years of age.

| **Metric** | **Recommended ideal level** |
| --- | --- |
| Smoking | Passive smoking. Parent never smoked or quit ≥12 months ago |
| Body mass index | Under the age- and sex-specific International Obesity Task Force IOTF threshold of childhood overweight (4) |
| Physical activity | ≥180 min/day outdoor games and walking (spring) or ≥162 min/day (other seasons) (2) |
| Healthy diet | ≥2 out of the following 3 items: 4.5 times of a fruit and vegetable portion/day, fish consumption ≥2 times/week, sugar-sweetened beverages <4 times a week |
| Blood pressure | Systolic and diastolic <90th height-age-sex-center specific percentile of EDEN population (5) |
| Fasting plasma glucose | <5.55 mmol/l (6) |
| Total cholesterol | <4.40 mmol/l (6) |

Overall cardiovascular health status: children with 5-6 metrics (excluding the smoking metric in main analysis) at the ideal level had an ideal cardiovascular health. When the smoking metric is considered (see STable 7 and STable 8), children with 5-7 metrics at the ideal level had an ideal cardiovascular health.

Behavioral cardiovascular health status: children with 0-1, 2 and 3 behavioral metrics (body mass index, physical activity, diet) at the ideal level had respectively a poor, intermediate and ideal behavioral cardiovascular health. When the smoking metric is considered (see eTable 7 and eTable 8), children with 0-1, 2 and 3-4 behavioral metrics at the ideal level had a respectively a poor, intermediate and ideal cardiovascular health.

Biological cardiovascular health status: children with 0-1, 2 and 3 biological metrics (blood pressure, total cholesterol and fasting glycaemia) at the ideal level had respectively a poor, intermediate and ideal biological cardiovascular health.

# STable2. Definition of ideal behavioural cardiovascular health metrics in the mother prior to pregnancy.

| **Metric** | **Recommended ideal level** |
| --- | --- |
| Smoking | No smoking in the three months prior to pregnancy |
| Body mass index | <25 kg/m² |
| Physical activity* | Frequently walking or bicycle or swimming or walk at work, or more than 150 minutes/week other sports and walk/bicycle to work |
| Healthy diet | ≥2 out of the following 3 items: 4.5 times of a fruit and vegetable portion/day, fish consumption ≥2 times/week, sugar-sweetened beverages <450 kcal per week |

Biological metrics of cardiovascular health including blood pressure, blood glucose and total cholesterol prior to pregnancy were not available.

*Physical activity collected at 24-28 weeks of amenorrhea was used as a proxy for the physical activity prior to pregnancy.

Women with 0-1, 2 and 3-4 behavioral metrics at the ideal level had respectively a poor, intermediate and ideal behavioral cardiovascular health.

# STable3. Early life characteristics of included and excluded children among those followed up until five years of age.

|  | **Included n=566** | **Excluded n=674** | **P value** |
| --- | --- | --- | --- |
| **Characteristics at birth** |  |  |  |
| Study center (Nancy vs Poitiers) | 326 (58) | 344 (51) | 0.021 |
| Male sex | 314 (56) | 346 (51) | 0.15 |
| Preterm | 29 (5) | 39 (6) | 0.61 |
| Gestational age, weeks (mean±SD) | 39±2 | 39±2 | 0.61 |
| Birth weight, grams (mean±SD) | 3288±518 | 3305±505 | 0.60 |
| **Characteristics after birth** |  |  |  |
| Breastfed | 420 (74) | 491 (73) | 0.55 |
| Time spent breastfeeding, months (mean±SD) | 5 (4) | 5 (4) | 0.76 |
| **Characteristics at three years of age** |  |  |  |
| Ideal level of blood pressure | 445 (84) | 452 (83) | 0.47 |
| Ideal level of body mass index | 487 (95) | 502 (94) | 0.51 |
| Ideal level of diet | 247 (48) | 249 (41) | 0.034 |
| Ideal consumption of fish | 275 (53) | 280 (47) | 0.028 |
| Ideal consumption of fruits and vegetables | 27 (5) | 38 (6) | 0.43 |
| Ideal consumption of sugar sweetened beverages | 441 (85) | 481 (80) | 0.022 |
| Ideal level of physical activity | 195 (38) | 222 (38) | 0.92 |
| Behavioral cardiovascular health* |  |  |  |
| *Poor* | 185 (38) | 223 (42) | 0.086 |
| *Intermediate* | 212 (44) | 234 (44) |  |
| *Ideal* | 90 (19) | 72 (14) |  |
| Time spent watching television daily |  |  |  |
| *≤30 minutes* | 138 (27) | 159 (27) | 0.65 |
| *>30 minutes to 1 hour* | 186 (36) | 198 (34) |  |
| *>1 hour* | 186 (36) | 226 (39) |  |
| Overnight sleep duration, hours (mean±SD) | 11±0.6 | 11±0.7 | 0.42 |
| **Characteristics at five years of age** |  |  |  |
| Age, years (mean±SD) | 5.59±0.14 | 5.59±0.17 | 0.80 |
| Weight, kg (mean±SD) | 20±3 | 20±3 | 0.13 |
| Height, cm (mean±SD) | 114±5 | 114±5 | 0.88 |
| Time spent watching television daily |  |  |  |
| *≤30 minutes* | 64 (12) | 79 (13) | 0.67 |
| *>30 minutes to 1 hour* | 169 (31) | 180 (30) |  |
| *>1 hour* | 319 (58) | 334 (56) |  |
| Overnight sleep duration, hours (mean±SD) | 11±0.5 | 11±0.5 | 0.19 |

Data are n (%) unless otherwise stated.

*Behavioral cardiovascular health status at 3 years of age: children with 0-1, 2 and 3 behavioral metrics (body mass index, physical activity, diet; the smoking metric is excluded in main analysis) at the ideal level had respectively a poor, intermediate and ideal behavioral cardiovascular health

**STable4. Parental characteristics of included and excluded children among those followed up until five years of age.**

|  | **Included n=566** | **Excluded n=674** | **P value** |
| --- | --- | --- | --- |
| **Sociodemographic factors** |  |  |  |
| Mother born in France | 549 (98) | 645 (96) | 0.056 |
| Household income, euros |  |  |  |
| *<2300* | 162 (29) | 187 (28) | 0.004 |
| *2300-3000* | 188 (33) | 178 (26) |  |
| *3000-3800* | 135 (24) | 166 (25) |  |
| *>3800* | 81 (14) | 143 (21) |  |
| Family education level |  |  |  |
| *High school* | 86 (15) | 86 (13) | 0.39 |
| *Secondary* | 98 (17) | 129 (19) |  |
| *Tertiary* | 382 (68) | 459 (68) |  |
| Family stimulation score | 17±2 | 17±2 | 0.38 |
| Age of mother at delivery | 30±5 | 30±5 | 0.51 |
| Age of father at delivery | 33±6 | 32±6 | 0.20 |
| **Father at inclusion** |  |  |  |
| Body mass index, kg/m^2^ (mean±SD) | 25.42±3.69 | 24.99±3.59 | 0.066 |
| **Mother prior to pregnancy** |  |  |  |
| Hypertension | 12 (2) | 18 (3) | 0.53 |
| Ideal level of body mass index | 403 (72) | 501 (74) | 0.40 |
| Ideal level of diet | 40 (7) | 59 (9) | 0.29 |
| Ideal consumption of fish | 226 (40) | 261 (39) | 0.64 |
| Ideal consumption of fruit and vegetables | 144 (26) | 168 (25) | 0.82 |
| Ideal consumption of sugar sweetened beverage | 294 (53) | 365 (55) | 0.46 |
| Ideal level of smoking | 399 (71) | 446 (67) | 0.13 |
| Ideal level of physical activity* | 366 (65) | 406 (60) | 0.13 |
| Ideal level of behavioral cardiovascular health^†^ | 201 (37) | 233 (35) | 0.63 |
| **Mother during pregnancy** |  |  |  |
| Depressive symptoms (CESD>23) | 39 (7) | 46 (7) | 0.96 |
| Alcohol consumption during first trimester |  |  |  |
| *0 glasses/week* | 300 (53) | 370 (55) | 0.79 |
| *]0-2[glasses/week* | 219 (39) | 252 (37) |  |
| *>2glasses/week* | 47 (8) | 52 (8) |  |
| Smoker | 113 (20) | 155 (23) | 0.20 |
| Gestational diabetes | 41 (7) | 49 (7) | 0.98 |
| Hypertension | 27 (5) | 34 (5) | 0.82 |
| **Mother when child five years of age** |  |  |  |
| Live alone | 35 (6) | 49 (8) | 0.300 |
| Depressive symptoms (CESD>23) | 52 (10) | 34 (6) | 0.010 |

Data are n (%) unless otherwise stated. CESD, French version of the Center for Epidemiologic Studies–Depression (8, 9). A score >=23 is indicative of a high likelihood of clinical depression.

*Physical activity collected at 24-28 weeks of amenorrhea was used as a proxy for physical activity prior to pregnancy.

^†^Behavioral cardiovascular health status in the mother prior to pregnancy: women with 0-1, 2 and 3-4 behavioral metrics (smoking, body mass index, physical activity, diet) at the ideal level had respectively a poor, intermediate and ideal behavioral cardiovascular health.

# STable5. Parental characteristics in children with and without ideal cardiovascular health (CVH) at five years age.

|  | **Ideal CVH* at age 5 n=195** | **Non ideal CVH at age 5 n=371** | **P value** |
| --- | --- | --- | --- |
| **Socio demographic factors** |  |  |  |
| Mother born in France | 188 (97) | 361 (99) | 0.33 |
| Household income, euros |  |  |  |
| *<2300* | 58 (30) | 104 (28) | 0.094 |
| *2300-3000* | 56 (29) | 132 (36) |  |
| *3000-3800* | 57 (29) | 78 (21) |  |
| *>3800* | 24 (12) | 57 (15) |  |
| Family education level |  |  |  |
| *High school* | 31 (16) | 55 (15) | 0.88 |
| *Secondary* | 35 (18) | 63 (17) |  |
| *Tertiary* | 129 (66) | 253 (69) |  |
| Family stimulation score | 17±2 | 17±2 | 0.89 |
| Age of mother at delivery, years | 30±5 | 30±5 | 0.29 |
| Age of father at delivery, years | 32±5 | 33±6 | 0.66 |
| **Father at inclusion** |  |  |  |
| Body mass index, kg/m^2^ (mean±SD) | 25.24 ±3.61 | 25.52± 3.73 | 0.51 |
| **Mother prior to pregnancy** |  |  |  |
| Hypertension | 5 (3) | 7 (2) | 0.56 |
| Ideal level of body mass index | 136 (71) | 267 (73) | 0.50 |
| Ideal level of diet | 15 (8) | 25 (7) | 0.63 |
| Ideal consumption of fish | 91 (47) | 135 (37) | 0.014 |
| Ideal consumption of fruit and vegetables | 61 (32) | 83 (23) | 0.015 |
| Ideal consumption of sugar sweetened beverage | 96 (50) | 198 (54) | 0.36 |
| Ideal level of physical activity^†^ | 131 (67) | 235 (63) | 0.36 |
| Ideal level of smoking | 138 (71) | 261 (70) | 0.92 |
| Ideal level of behavioral cardiovascular health‡ | 70 (37) | 131 (36) | 0.84 |
| **Mother during pregnancy** |  |  |  |
| Alcohol consumption |  |  |  |
| *0 glasses/week* | 110 (56) | 190 (51) | 0.20 |
| *]0-2[glasses/week* | 66 (34) | 153 (41) |  |
| *>=2 glasses/week* | 19 (10) | 28 (8) |  |
| Smoker | 36 (19) | 77 (21) | 0.48 |
| Depressive symptoms (CESD>23) | 12 (6) | 27 (7) | 0.61 |
| Hypertension | 9 (5) | 18 (5) | 0.90 |
| Gestational diabetes | 15 (8) | 26 (7) | 0.77 |
| **Mother when child five years of age** |  |  |  |
| Live alone | 11 (6) | 24 (7) | 0.69 |
| Depression symptoms (CESD>23) | 18 (9) | 34 (9) | 0.99 |

Data are n (%) unless otherwise stated. CESD, French version of the Center for Epidemiologic Studies–Depression (8, 9). A score >=23 is indicative of a high likelihood of clinical depression.

*Children with 5-6 metrics (body mass index, physical activity, diet, blood pressure, glycemia and total cholesterol; excluding the smoking metric in main analysis) at the ideal level had an ideal cardiovascular health.

^†^Physical activity collected at 24-28 weeks of amenorrhea was used as a proxy for physical activity prior to pregnancy.

^‡^Behavioral cardiovascular health status in the mother prior to pregnancy: women with 0-1, 2 and 3-4 behavioral metrics (smoking, body mass index, physical activity, diet) at the ideal level had respectively a poor, intermediate and ideal behavioral cardiovascular health.

# STable6. Determinants of number of cardiovascular health metrics* at ideal level in children at five years of age.

| Determinants | | Model 1 | Model 2 | Model 3 | Model 4 |
| --- | --- | --- | --- | --- | --- |
|  | | **OR (95%CI)** | **OR (95%CI)** | **OR (95%CI)** | **OR (95%CI)** |
| Level 1 | Study center, Nancy vs Poitiers | 0.94 (0.68; 1.32) | 0.84 (0.58; 1.22) | 0.84 (0.57; 1.24) | 0.77 (0.49; 1.19) |
|  | Age of mother, years | 1.02 (0.97; 1.07) | 1.01 (0.96; 1.06) | 1.01 (0.96; 1.06) | 1.01 (0.96; 1.08) |
|  | Age of father, years | 0.98 (0.94; 1.02) | 1.00 (0.96; 1.05) | 1.00 (0.96; 1.04) | 0.99 (0.95; 1.04) |
|  | Family educational level |  |  |  |  |
|  | *Secondary vs high school* | 1.07 (0.59; 1.93) | 1.32 (0.70; 2.50) | 1.28 (0.67; 2.44) | 1.23 (0.56; 2.67) |
|  | *Tertiary vs high school* | 0.81 (0.50; 1.30) | 0.84 (0.50; 1.42) | 0.78 (0.46; 1.34) | 0.77 (0.40; 1.50) |
|  | Mother living alone when child 5 years of age | 0.78 (0.40; 1.53) | 0.84 (0.42; 1.69) | 0.82 (0.41; 1.66) | 0.60 (0.25; 1.41) |
|  | Mother born in France | 1.03 (0.56; 1.90) | 0.70 (0.33; 1.47) | 1.11 (0.48; 2.55) | 0.89 (0.30; 2.67) |
| Level 2 | Hypertension prior to pregnancy |  | 2.38 (0.65; 8.80) | 2.31 (0.62; 8.56) | 2.78 (0.66; 11.73) |
|  | Behavioral cardiovascular health prior to pregnancy^†^ |  |  |  |  |
|  | *Intermediate vs poor* |  | 1.06 (0.65; 1.71) | 1.18 (0.72; 1.94) | 1.58 (0.90; 2.78) |
|  | *Ideal vs poor* |  | 1.15 (0.68; 1.94) | 1.25 (0.73; 2.12) | 1.51 (0.83; 2.75) |
|  | Comorbidities that developed during pregnancy‡ |  | 1.23 (0.70; 2.18) | 1.29 (0.72; 2.28) | 1.25 (0.65; 2.42) |
|  | Depressive symptoms during pregnancy, CESD>=23 |  | 0.82 (0.41; 1.65) | 0.89 (0.44; 1.80) | 1.05 (0.45; 2.42) |
|  | Smoked during pregnancy |  | 1.06 (0.66; 1.69) | 1.08 (0.67; 1.75) | 0.91 (0.53; 1.58) |
|  | Consumed alcohol during pregnancy |  |  |  |  |
|  | *0-2 vs no glasses/week* |  | 1.42 (0.95; 2.13) | 0.74 (0.50; 1.09) | 0.63 (0.41; 0.98) |
|  | *≥2 vs no glasses/week* |  | 1.64 (0.98; 2.76) | 1.44 (0.74; 2.82) | 1.52 (0.72; 3.19) |
|  | Primiparous |  | 1.30 (0.88; 1.91) | 1.25 (0.85; 1.84) | 1.19 (0.77; 1.83) |
|  | Father ideal body mass index |  | 1.31 (0.92; 1.85) | 1.35 (0.95; 1.93) | 1.39 (0.92; 2.08) |
| Level3 | Sex, boy vs girl |  |  | 1.60 (1.13; 2.27) | 1.81 (1.22; 2.68) |
|  | Gestational age |  |  | 1.08 (0.97; 1.20) | 1.03 (0.91; 1.17) |
|  | Birth weight z-score |  |  | 0.99 (0.84; 1.18) | 0.90 (0.74; 1.09) |
|  | Breastfed vs not breastfed |  |  | 1.21 (0.80; 1.83) | 1.45 (0.90; 2.34) |
| Level 4 | Behavioral cardiovascular health at 3 years¶ |  |  |  |  |
|  | *Intermediate vs poor* |  |  |  | 1.13 (0.72; 1.77) |
|  | *Ideal vs poor* |  |  |  | 2.79 (1.58; 4.93) |
|  | Overnight sleep duration, hours |  |  |  | 0.72 (0.53; 0.99) |
|  | Watching television |  |  |  |  |
|  | *≤30 minutes vs 30 minutes to 1 hour/day* |  |  |  | 1.84 (1.11; 3.03) |
|  | *>1 hour vs 30 minutes to 1 hour/day* |  |  |  | 1.21 (0.76; 1.95) |

Multivariate ordinal logistic regression of number of cardiovascular health metrics at ideal level performed by adding several variables in blocks. Model 1 is adjusted for socio-demographic factors, model 2 is adjusted for model 1 + the mothers pregnancy data, model 3 is adjusted for model 2 + birth data and model 4 is adjusted for model 3 + relevant cardiovascular health data of the children at three years of age. CESD, French version of the Center for Epidemiologic Studies–Depression (8, 9). A score >=23 is indicative of a high likelihood of clinical depression. Birth weight z score was defined based on gestation age, height, maternal weight and parity.

*The numbers of ideal metrics range 0 to 6 and include body mass index, physical activity, diet, blood pressure, glycemia and total cholesterol; the smoking metric is considered in eTable7 and eTable8.

^†^Hypertension or gestational diabetes.

‡Children with 0-1, 2 and 3 behavioral metrics (body mass index, physical activity, diet) at the ideal level had respectively a poor, intermediate and ideal behavioral cardiovascular health.

¶Women with 0-1, 2 and 3-4 behavioral metrics (additionally including smoking) at the ideal level had respectively a poor, intermediate and ideal behavioral cardiovascular health.

Due to missing confounding data in each model, the sample size varied as follows: model 1 n=485, model 2 = n=447 model 3 n=442, model 4 n=361.

**STable7. Determinants of ideal cardiovascular health status in children at five years of age after including passive smoking* in the definition of cardiovascular health.**

| **Determinants** | | **Model 1** | **Model 2** | **Model 3** | **Model 4** |
| --- | --- | --- | --- | --- | --- |
|  | | OR (95%CI) | OR (95%CI) | OR (95%CI) | OR (95%CI) |
| **Level 1** | Study center (Nancy vs Poitiers) | 0.75 (0.53; 1.07) | 0.70 (0.47; 1.03) | 0.71 (0.47; 1.07) | 0.73 (0.45; 1.18) |
|  | Age of mother (years) | 1.03 (0.98; 1.08) | 1.02 (0.96; 1.07) | 1.01 (0.96; 1.07) | 1.04 (0.97; 1.11) |
|  | Age of father (years) | 0.99 (0.95; 1.03) | 1.00 (0.96; 1.05) | 1.00 (0.95; 1.05) | 1.00 (0.94; 1.05) |
|  | Family educational level |  |  |  |  |
|  | *Secondary vs high school* | 1.29 (0.70; 2.37) | 1.19 (0.60; 2.37) | 1.18 (0.58; 2.37) | 1.07 (0.46; 2.54) |
|  | *Tertiary vs high school* | 1.19 (0.72; 1.96) | 0.92 (0.52; 1.65) | 0.85 (0.47; 1.54) | 0.85 (0.41; 1.77) |
|  | Mother living alone when child 5 years of age | 0.48 (0.23; 0.97) | 0.41 (0.19; 0.89) | 0.38 (0.17; 0.85) | 0.30 (0.11; 0.82) |
|  | Mother born in France | 1.73 (0.79; 3.78) | 1.22 (0.53; 2.84) | 1.90 (0.62; 5.76) | 1.55 (0.47; 5.14) |
| **Level 2** | Hypertension prior to pregnancy |  | 1.46 (0.36; 5.99) | 1.46 (0.35; 6.06) | 1.91 (0.35; 10.43) |
|  | Behavioral cardiovascular health prior to pregnancy^†^ |  |  |  |  |
|  | *Intermediate vs poor* |  | 1.08 (0.65; 1.80) | 1.07 (0.63; 1.81) | 1.22 (0.66; 2.24) |
|  | *Ideal vs poor* |  | 1.34 (0.77; 2.34) | 1.38 (0.78; 2.44) | 1.46 (0.76; 2.82) |
|  | Comorbidities that developed during pregnancy‡ |  | 1.51 (0.81; 2.82) | 1.58 (0.83; 3.00) | 1.33 (0.63; 2.81) |
|  | Depressive symptoms during pregnancy, CESD>=23 |  | 0.59 (0.28; 1.25) | 0.64 (0.30; 1.38) | 0.64 (0.25; 1.63) |
|  | Smoking during pregnancy |  | 0.43 (0.26; 0.72) | 0.42 (0.25; 0.70) | 0.35 (0.19; 0.64) |
|  | Alcohol consumption during pregnancy |  |  |  |  |
|  | *0-2 vs no glasses/week* |  | 0.88 (0.59; 1.31) | 0.89 (0.59; 1.34) | 0.76 (0.48; 1.23) |
|  | *≥2 vs no glasses/week* |  | 1.34 (0.65; 2.77) | 1.39 (0.65; 2.95) | 1.33 (0.58; 3.03) |
|  | Primiparous |  | 1.05 (0.69; 1.58) | 1.02 (0.67; 1.55) | 1.09 (0.67; 1.77) |
|  | Father ideal body mass index |  | 1.20 (0.82; 1.74) | 1.23 (0.84; 1.80) | 1.21 (0.78; 1.89) |
| **Level 3** | Sex (boy vs girl) |  |  | 1.84 (1.27; 2.68) | 2.34 (1.51; 3.61) |
|  | Gestational age |  |  | 1.05 (0.94; 1.17) | 1.04 (0.91; 1.20) |
|  | Birth weight z-score |  |  | 0.97 (0.81; 1.17) | 0.90 (0.73; 1.11) |
|  | Breastfed vs not breastfed |  |  | 1.26 (0.81; 1.97) | 1.52 (0.91; 2.53) |
| **Level 4** | Behavioral cardiovascular health at 3 years of age¶ |  |  |  |  |
|  | *Intermediate vs poor* |  |  |  | 0.99 (0.61; 1.61) |
|  | *Ideal vs poor* |  |  |  | 2.14 (1.11; 4.11) |
|  | Overnight sleep duration (hours) |  |  |  | 0.92 (0.65; 1.31) |
|  | Watching television |  |  |  |  |
|  | *≤30 minutes vs 30 minutes to 1 hour/day* |  |  |  | 1.89 (1.08; 3.30) |
|  | *>1 hour vs 30 minutes to 1 hour/day* |  |  |  | 1.25 (0.74; 2.08) |

Multivariate logistic regression of ideal cardiovascular health status compared to non-ideal cardiovascular health status were performed by adding several variables in blocks. Model 1 is adjusted for socio-demographic factors, model 2 is adjusted for model 1 + the mothers pregnancy data, model 3 is adjusted for model 2 + birth data and model 4 is adjusted for model 3 + relevant cardiovascular health data of the children at three years of age. CESD, French version of the Center for Epidemiologic Studies–Depression (8, 9). A score >=23 is indicative of a high likelihood of clinical depression. Birth weight z score was defined based on gestation age, height, maternal weight and parity. Refer to footnote of Table S1 for the definition of overall and behavioral CVH at age 3 and 5 when including the smoking metric.

*Children exposed to parental smoking at age five

^†^Women with 0-1, 2 and 3-4 behavioral metrics (additionally including smoking) at the ideal level had respectively a poor, intermediate and ideal behavioral cardiovascular health.

‡Hypertension or gestational diabetes.

¶Children at 3 years of age with 0-1, 2 and 3 behavioral metrics (body mass index, physical activity, diet) at the ideal level had respectively a poor, intermediate and ideal behavioral cardiovascular health.

Due to missing confounding data in each model, the sample size varied as follows: model 1 n=574, model 2 = n=523 model 3 n=518, model 4 n=419.

# STable8. Cross-sectional association between cardiovascular health and intelligence quotient (IQ) in children at five years of age after including passive smoking* in the definition of cardiovascular health.

|  | **Total IQ** | | **Verbal IQ** | | **Performance IQ** | | |
| --- | --- | --- | --- | --- | --- | --- | --- |
|  | **β (95%CI)** | **P value** | **β (95%CI)** | **P value** | **β (95%CI)** | **P value** |  |
| *Overall cardiovascular health* | | | | | | | |
| Ideal vs. non ideal status | 1.50 (-0.87 ; 3.88) | 0.21 | 1.99 (-0.50 ; 4.49) | 0.12 | 0.36 (-2.18 ; 2.90) | 0.78 |  |
| Number of ideal metrics  (range 0 to 7): per 1 unit increase | 1.13 (-0.10 ; 2.37) | 0.074 | 1.12 (-0.18 ; 2.42) | 0.092 | 0.85 (-0.48 ; 2.18) | 0.21 |  |
| *Behavioral cardiovascular health* | | | | | | | |
| Ideal vs. non ideal status | 1.15 (-0.63 ; 2.94) | 0.20 | 1.77 (-0.10 ; 3.65) | 0.064 | 0.16 (-1.74 ; 2.07) | 0.86 |  |
| Number of ideal metrics  (range 0 to 4): per 1 unit increase | 0.63 (-0.45 ; 1.71) | 0.26 | 1.30 (0.16 ; 2.44) | 0.026 | -0.15 (-1.30 ; 1.01) | 0.80 |  |
| *Biological cardiovascular health*^†^ | | | | | | | |
| Ideal vs. non ideal status | 1.62 (-0.62; 3.86) | 0.16 | -0.18 (-2.54; 2.18) | 0.88 | 2.98 (0.64; 5.32) | 0.013 |  |
| Number of ideal metrics  (range 0 to 3): per 1 unit increase | 1.62 (-0.19; 3.42) | 0.080 | 0.23 (-1.67; 2.13) | 0.81 | 2.48 (0.59; 4.36) | 0.010 |  |

Multivariate linear regression analysis adjusted for study center, maternal age, maternal pre pregnancy body mass index, depression during pregnancy, smoking and alcohol consumption during pregnancy, parity, breastfeeding, child’s sex, birth weight z score, gestational age, parental education, household income, family stimulation score and time spent watching television at age five.

Age-adjusted composite scores for verbal, performance and total intelligence quotient (IQ) were calculated based on information obtained from the French version of the Wechsler Preschool and Primary Scale of Intelligence (11).

*Children exposed to parental exposure at age five.

^†^ Results for biological cardiovascular health are the same as those reported in Table 3 as the addition of the smoking metric only affects behavioral cardiovascular health.

Overall cardiovascular health status: children with 5-7 metrics at the ideal level had an ideal cardiovascular health.

Behavioral cardiovascular health status: children with 3-4 behavioral metrics (smoking, body mass index, physical activity, diet) at the ideal level had an ideal behavioral cardiovascular health.

Biological cardiovascular health status: children with 3 biological metrics (blood pressure, total cholesterol and fasting glycaemia) at the ideal level had an ideal biological cardiovascular health.

Due to missing confounding data the sample size varied between 403 and 564 for overall CVH, between 493 and 498 for biological CVH and between 731 and 892 for behavioral CVH.

# Supplementary Material References

1. Heude B, Forhan A, Slama R, Douhaud L, Bedel S, Saurel-Cubizolles M-J, et al. Cohort Profile: The EDEN mother-child cohort on the prenatal and early postnatal determinants of child health and development. International Journal of Epidemiology. 2016;45(2):353-63.

2. Saldanha-Gomes C, Heude B, Charles M, de Lauzon-Guillain B, Botton J, Carles S, et al. Prospective associations between energy balance-related behaviors at 2 years of age and subsequent adiposity: the EDEN mother–child cohort. International Journal of Obesity. 2017;41(1):38-45.

3. EDEN Mother-Child Cohort Study Group. Dietary patterns track from infancy to preschool age: cross-sectional and longitudinal perspectives. The Journal of nutrition. 2015;145(4):775-82.

4. Cole TJ, Lobstein T. Extended international (IOTF) body mass index cut‐offs for thinness, overweight and obesity. Pediatric obesity. 2012;7(4):284-94.

5. Perng W, Francis EC, Schuldt C, Barbosa G, Dabelea D, Sauder KA. Pre-and Perinatal Correlates of Ideal Cardiovascular Health (ICVH) During Early Childhood: A Prospective Analysis in the Healthy Start Study. The Journal of Pediatrics. 2021.

6. Labarthe DR, Goldstein LB, Antman EM, Arnett DK, Fonarow GC, Alberts MJ, et al. Evidence-based policy making: assessment of the American Heart Association’s strategic policy portfolio: a policy statement from the American Heart Association. Circulation. 2016;133(18):e615-e53.

7. Baecke JA, Burema J, Frijters JE. A short questionnaire for the measurement of habitual physical activity in epidemiological studies. The American journal of clinical nutrition. 1982;36(5):936-42.

8. Fuhrer R, Rouillon F. La version française de l’échelle CES-D (Center for Epidemiologic Studies-Depression Scale). Description et traduction de l’échelle d’autoévaluation. Psychiatry and Psychobiology. 1989;4(3):163-6.

9. Radloff LS. The CES-D scale: A self-report depression scale for research in the general population. Applied psychological measurement. 1977;1(3):385-401.

10. Bradley RH, Caldwell BM, Rock SL, Hamrick HM, Harris P. Home observation for measurement of the environment: Development of a home inventory for use with families having children 6 to 10 years old. Contemporary Educational Psychology. 1988;13(1):58-71.

11. Gordon B. Test Review: Wechsler, D.(2002). The Wechsler Preschool and Primary Scale of Intelligence, (WPPSI-III). San Antonio, TX: The Psychological Corporation. Canadian Journal of School Psychology. 2004;19(1-2):205-20.
